# Consumption of hookahs, e-cigarettes, and classic cigarettes and the impact on medically assisted reproduction treatment

**DOI:** 10.1038/s41598-024-60251-y

**Published:** 2024-04-26

**Authors:** Tom Trapphoff, Carolin Ontrup, Sonja Krug, Stefan Dieterle

**Affiliations:** 1 Fertility Center Dortmund, Olpe 19, 44135 Dortmund, Germany; 2 Division of Reproductive Endocrinology and Infertility, Department of Obstetrics and Gynecology, University Witten/Herdecke, 44135 Dortmund, Germany

**Keywords:** Medical research, Risk factors

## Abstract

Smoking of classic cigarettes has been well-established as a health risk factor, including cardiovascular, neurological, and pulmonary diseases. Adverse effects on human reproduction have also been shown. Smokers are assumed to have a significantly lower chance of pregnancy, however, the impact of smoking on medically assisted reproduction (MAR) treatment outcomes is controversial. Moreover, smoking habits have changed during the last decades since e-cigarettes and hookahs, or water pipes, have become very popular, yet little is known regarding vaping or hookah-smoking patients undergoing MAR treatments. This prospective study aimed to examine the presence of benzo[a]pyrene, nicotine, and its main metabolite, cotinine, in human follicular fluid (FF) in non-smoking, smoking, and vaping/hookah-smoking patients and to evaluate the impact on female fertility. Human FF samples were collected from 320 women subjected to intracytoplasmic sperm injection (ICSI) cycles due to male subfertility. Gas chromatography combined with mass spectrometry was used to analyse the presence of benzo[a]pyrene, nicotine, and cotinine. A questionnaire was provided to assess patient consumption behaviour and to identify (1) non-smoking patients, (2) patients who consumed cigarettes, and (3) patients with exclusive consumption of e-cigarettes or hookahs. Data were analysed using linear and logistic regression, Fisher’s exact test, and the Mann–Whitney U Test. Nicotine was present in 22 (6.8%) and cotinine in 65 (20.3%) of the 320 samples. The nicotine and cotinine concentrations per sample ranged from 0 to 26.3 ng/ml and 0–363.0 ng/ml, respectively. Benzo[a]pyrene was not detectable in any of the samples analysed. Nicotine and cotinine were also present in the FF of patients with exclusive consumption of e-cigarettes or hookahs. The clinical pregnancy rate, fertilization and maturation rates, and number of oocytes per oocyte pick-up were not statistically significantly different between non-smoking, smoking, or vaping/hookah-smoking patients. Smoking and the accumulation of smoking toxins in the FF have no impact on the outcome of MAR treatments—neither the clinical pregnancy rate, maturation and fertilization rates, nor the number of retrieved oocytes were affected. For the first time, nicotine and cotinine were quantified in the FF of patients exclusively vaping e-cigarettes or smoking hookahs. Since vaping liquids and hookah tobaccos contain potentially harmful substances, other adverse effects cannot be excluded.

**Trial registration** ClinicalTrials.gov Identifier: NCT03414567.

## Introduction

Smoking, a well-established risk factor for general health, is linked to cardiovascular, neurological, and pulmonary diseases^1–3^. Adverse effects on human reproduction have been reported in the last decades, including a significantly higher risk of preterm birth, miscarriage, and malformations, as well as reduced implantation and live birth rates^4–7^. Similar effects have been described for passive smokers^8,9^. Overall, smokers exhibit a significantly lower likelihood of achieving a successful pregnancy^4,10^.

The burning of tobacco products and additives, such as menthol, glycerine, and sorbic acid, produces more than 4000 chemicals—many of which have neurotoxic and carcinogenic characteristics^11–13^. Known neurotoxic substances include nicotine and cotinine, its main metabolite^14,15^. Due to its toxic characteristics, nicotine is classified as ‘acute toxic’ according to European Chemical Agency (ECHA) with an oral toxicity LC_50_ (lethal concentration) of 3.3 mg nicotine per kg body weight for mice and an acute inhalation toxicity of 2.3 mg nicotine/L for 20 min for rats. For humans, acute toxicity estimates (ATE) were 3.3 mg per kg body weight for acute oral toxicity and 0.25 mg/L for acute inhalation toxicity^65^. Nicotine is mainly metabolised by different liver enzymes including flavin-containing monooxygenase, Cytochrome P450 2A6 oxidase system, or UDP-glucuronosyltransferase into more than 20 (intermediate) metabolites and primarily excreted in the urine^18,66^. In humans, on average 70–80% of nicotine is metabolised through the cotinine pathway^66^. The half-life of nicotine is about two hours in body fluids, while cotinine has a half-life of around 16 h^16–18^. Thus, cotinine is the most widely used biomarker of nicotine intake.

Additionally, the incomplete combustion of tobacco products produces the carcinogen benzo[a]pyrene^19,20^. The long-term and immediate effects of nicotine and benzo[a]pyrene exposure on female reproduction have been explored using human cell lines, different animal models including rodents and Rhesus monkeys, and in clinical studies. The effect of nicotine is to lower progesterone levels by inhibiting the progesterone synthesis and by reduction of cell growth, to limit oestrogen biosynthesis, to decrease peripheral blood flow, and to impair fallopian tube and uterus contractility^21–23,67–69^. Benzo[a]pyrene hampers cell proliferation and oestrogen biosynthesis and is linked to DNA damage^24,25^.

Besides classic cigarette smoking, consumption of electronic cigarettes (e-cigarettes) and smoking of water pipes or hookahs have increased rapidly in recent years and are popular among adolescent males and females^26,27^. In 2021, more than 82 million people worldwide were estimated to be regularly consuming e-cigarettes^26^. Since heating, combustion, and inhalation are different compared with smoking classic cigarettes, many consumers particularly regard e-cigarettes as an alternative to classic cigarette consumption. However, e-cigarette vaping liquids, even nicotine-free ones, may affect health and human reproduction, especially since vaping liquids, hookah tobacco, and aerosols contain several potentially harmful substances, including glycerine, formaldehyde, acetaldehyde, methylglyoxal, benzaldehyde, and endocrine-disrupting chemicals like phthalates or polybrominated diphenyl ethers, respectively^28–32,70^. Overall, the effects of exposure to e-cigarette vapours and hookah smoking on human reproduction are poorly understood.

The influence of smoking on the outcomes of medically assisted reproduction (MAR) treatments has been studied and debated for over 40 years; however, the current literature affords controversial results. While some studies reported adverse effects for smokers at different levels^33–35^, others found no correlation between smoking and MAR outcomes^36–38^. However, quality and quantity of smoking differ individually and must be taken into account when assessing the influence of smoking on the outcome of MAR treatment. Nicotine, cotinine, or benzo[a]pyrene accumulation in ovarian tissue or follicular fluid (FF) has not been sufficiently investigated, particularly their effects on MAR treatment outcomes^8,38–40^. Data regarding the consumption of e-cigarettes and smoking of water pipes or hookahs, to the best of our knowledge, have not been reported as yet.

In this prospective study, gas chromatography coupled with mass spectrometry (GC–MS) was used to analyse nicotine, cotinine, and benzo[a]pyrene accumulation in the FF of patients undergoing ICSI treatment. Patient consumption behaviour was assessed via a questionnaire to identify *i*) non-smoking patients, *ii*) patients who consumed classic cigarettes, and *iii*) patients with exclusive consumption of e-cigarettes or hookahs. The data were used to analyse the impact of smoking/vaping products in the FF on MAR outcomes.

## Material and methods

### Study population

Our study population in this prospective cohort study comprised 320 women subjected to ICSI treatment due to male subfertility. All men included had reduced semen parameters (oligozoospermia, asthenozoospermia and/or teratozoospermia) according to World Health Organization (WHO) criteria for examination and processing of human semen^71^. Women presenting endometriosis or polycystic ovaries were excluded. Human FF samples were collected during oocyte pick-up (OPU) at the Fertility Center Dortmund (Germany) from 2018 to 2023. Written informed consent was given by all patients before MAR treatment. The ethics committee of the University of Witten/Herdecke reviewed and approved the study (#193/2017; Germany). All methods were performed in accordance with the relevant guidelines and regulations.

The following parameters were assessed: serum oestradiol concentration (E2; pg/ml); Anti-Müllerian hormone level (AMH; ng/ml); gonadotropin dosage for ovarian stimulation (International units, IU); the numbers of oocytes retrieved, metaphase II (MII) oocytes, oocytes without pronuclei (0PN), one pronucleus (1PN), two (2PN), or more than two pronuclei (≥ 3PN); day of embryo transfer; number of transferred embryos; presence of a clinical pregnancy demonstrated by ultrasound (intrauterine cavity); age; and body mass index (BMI). Serum oestradiol concentrations and Anti-Müllerian hormone levels were analysed with immunoassay Atellica IM 1300 (Siemens Healthcare) platform.

The clinical pregnancy rate is the number of clinical pregnancies per number of OPUs. The oocyte maturation rate is the number of MII oocytes per number of retrieved oocytes. The fertilisation rate is the number of 2PN per number of MII oocytes.

A multiple-choice questionnaire was provided to evaluate the quality and quantity of consumer behaviour. Patients were divided into three groups: (1) non-smoking patients, (2) patients with consumption of classic cigarettes (with or without additional consumption of e-cigarettes and/or hookahs), and (3) patients with exclusive consumption of e-cigarettes and/or hookahs (without additional consumption of classic cigarettes).

### Sample collection and chemical analysis

All samples were collected during routine MAR treatment. Cumulus-oocyte complex aspiration was performed by ultrasound-guided OPU^72^. Oocytes underwent processing for further ICSI treatment^73^. The remaining FF (including cellular debris) without cumulus-oocyte complexes were directly transferred to sterile glass tubes and frozen at − 20 °C immediately. FF collection was limited to the first two follicles punctured.

Frozen samples were adjusted to room temperature before workup. All samples were spiked with nicotine-^13^CD_3_, cotinine-D_3_, and benzo[a]pyrene-D_12_ as internal standards. Samples were alkalised with 25% ammonium hydroxide to pH 10–11, extracted using chloroform, and evaporated at 45 °C in an N_2_ atmosphere. The residue was taken up in acetonitrile before performing GC–MS analysis on a nonpolar phase. For quantification, different concentrations of nicotine, cotinine, and benzo[a]pyrene standard solutions were prepared to ascertain their respective limits of detection (LOD) and quantification (LOQ).

GC–MS was carried out with the Agilent 8890 Gas Chromatograph system combined with an Agilent 5977B mass spectrometer. Compounds were separated on Agilent HP-5 ms Ultra Inert capillary columns (15 m, 0.25 mm id 0.25 µm film thickness) with a 0.5 µl injection volume. Retention times, target, and qualifier ions for all analytes are shown in Supplementary Table I. To ensure quality control, each measurement included standard solutions of known concentrations, solvent blanks, and samples of all reagents used. To control for any contamination during the sampling process or storage, all medical devices and vessels were analysed for any distorting substances before initiating the study.

### Statistics

This study was designed to assess the hypothesis that smoking of classic cigarettes or consumption of e-cigarettes and/or hookahs will negatively correlate with the outcome of a MAR treatment. The primary endpoint of this study is the clinical pregnancy rate per OPU. Patient base characteristics and clinical history include female and male ages (years), BMI (kg/m^2^), serum oestradiol concentration (pg/ml), AMH (ng/ml), gonadotropin dosage for ovarian stimulation (IU), previous OPU cycles, day of embryo transfer, and number of transferred embryos, which are presented as means ± standard deviations (SD). Statistical analysis of patient base characteristics and clinical history was conducted with the Mann–Whitney U Test. Clinical parameters are presented as means with 95% confidence intervals (95%CI) using Fisher's exact test and the Mann–Whitney U Test. The concentrations of nicotine, cotinine, and benzo[a]pyrene are presented as means ± SD in ng/ml FF. Distributions of all analyte concentrations were verified with the Shapiro–Wilk test. For correlation analyses of two variables, single linear regression models including the correlation coefficient *r* and ANOVA test were applied. For multiple linear regression analyses, the coefficient of determination *R*^2^ and ANOVA test were used. Single and multinomial binary logistic regression models were applied to test for FF contamination levels and clinical pregnancy rates per OPU, including Nagelkerke *R*-square (*R*_N_^2^), Goodness of fit test, odds ratio of correlation, and 95%CI. *P*-values < 0.05 were considered statistically significant.

An initial power calculation was carried out to compare non-nicotine consumers and nicotine consumers (including classic cigarettes, e-cigarettes and hookahs). Based on data from the German Robert Koch Institute and Federal Statistical Office of Germany (DSTATIS)^81^, it was assumed that approximately 50% of women of childbearing age regularly or occasionally consume nicotine products (including classic cigarettes, e-cigarettes and hookahs). With an equal distribution between both groups, a test power of 0.8, an alpha of 0.05 and a relative decrease in the pregnancy rate of 40% for nicotine consumers, the calculated sample size was *n* = 150 for each group. During sample collection it became clear that only about one third of our patients were regular/occasional nicotine consumers (including an unexpected high proportion of vaping patients and/or hookahs consumers). After statistical consultation, the sample size was increased to *n* = 320 and the study population was divided into three groups: Group 1 (non-smoking patients), Group 2 (smokers with or without additional e-cigarette and/or hookah consumption) and Group 3 (exclusive e-cigarette and/or hookah consumption). Thus, a post-hoc power analysis was done for the clinical pregnancy rate per oocyte pick-up for each group (dichotomous endpoint, alpha 0.05). The post-hoc power was 0.08 for Group 2 (smoker) and 0.03 for Group 3 (e-cigarettes and/or hookahs).

### Ethics approval and consent to participate

The study was reviewed and approved by the ethics committee of the University of Witten/Herdecke (#193/2017; Germany). Written informed consent sheets were given by all patients before MAR treatment.

## Results

This study included a total of 320 women subjected to ICSI treatment due to male subfertility. According to the questionnaire, three patient groups were established: 217 were assigned to Group 1 (non-smoking patients), 61 to Group 2 (smokers with or without additional e-cigarette and/or hookah consumption), and 42 to Group 3 (exclusive e-cigarette and/or hookah consumption).

Regarding patient base characteristics and clinical history, which included average female and male ages, BMI, average serum oestradiol concentration before ovulation (in pg/ml), AMH prior to MAR treatment (in ng/ml), gonadotropin dosage for ovarian stimulation (in International Units), average number of previous OPU, day of embryo transfer, and number of transferred embryos, no significant differences were found between all groups (Table 1).

**Table 1:** Patient base characteristics and clinical history of all 320 patients in Group 1 (non-smokers), Group 2 (smokers), and Group 3 (e-cigarettes and/or hookahs).

	Group 1 Non-smokers	Group 2 Smokers		Group 3 E-cigarettes and/or hookahs	
			*p*		*p*
OPUs [*n*]	217	61		42	
Female age [Ø] ± SD	33.5 ± 3.4	32.5 ± 3.7	n.s.	31.6 ± 3.9	n.s.
Male age [Ø] ± SD	37.0 ± 5.7	36.1 ± 5.5	n.s.	35.2 ± 5.3	n.s.
BMI [Ø] ± SD	26.5 ± 5.6	28.0 ± 6.7	n.s.	25.9 ± 6.2	n.s.
E2 before ovulation [Ø] pg/ml ± SD	2219.5 ± 1275.8	2152.9 ± 1123.5	n.s.	2383.5 ± 1082.2	n.s.
AMH [Ø] ng/ml ± SD	4.3 ± 2.5	4.3 ± 2.9	n.s.	4.8 ± 2.8	n.s.
Gonadotropin dose [Ø] IU ± SD	1943.9 ± 682.2	2068.5 ± 761.5	n.s.	1941.5 ± 799.3	n.s.
Previous OPUs [Ø] ± SD	2.0 ± 1.7	1.8 ± 1.6	n.s.	1.5 ± 1.3	n.s.
Day of embryo transfer [Ø] ± SD	3.5 ± 1.1	3.3 ± 1.1	n.s.	3.4 ± 1.1	n.s.
Embryos transferred [Ø] ± SD	1.9 ± 0.4	1.9 ± 0.3	n.s.	1.9 ± 0.4	n.s.

Mann–Whitney U Test was used for statistical analysis between control (non-smokers) and study groups (smokers and E-cigarettes and/or hookahs, respectively).

OPU, Oocyte pick-up; BMI, Body Mass Index; E2, oestradiol; AMH, Anti-Müllerian hormone; IU, International units; SD, standard deviation; n.s., not significant.

Basic characteristics of consumer behaviour according to the questionnaire for non-smokers (Group 1), smokers (Group 2), and vaping and/or hookah consuming patients (Group 3) are shown in Table 2. For Group 1 (non-smokers), 71 out of 217 (32.7%) patients quit smoking months/years before MAR treatment and 27 out of 217 (12.4%) were passive smokers with a regularly smoking partner. 119 patients never smoked or vaped regularly or occasionally (54.9%).

**Table 2:** Basic characteristics of consumer behaviour according to the questionnaire of all 320 patients for non-smokers (Group 1), smokers (Group 2), and vaping and/or hookah consuming patients (Group 3).

Group/parameter	
Group 1 Non-smokers [*n*]	217
Of that patients who quit smoking months/years before MAR treatment [*n*]/[%]	71/32.7%
Of that patients with a regularly smoking partner [*n*]/[%]	27/12.4%
Group 2 Smokers [*n*]	61
Of that patients who exclusively smoke classic cigarettes [*n*]/[%]	48/78.7%
Of that patients who smoke classic cigarettes and consume regularly e-cigarettes [*n*]/[%]	3/4.9%
Of that patients who smoke classic cigarettes and consume hookahs [*n*]/[%]	10/16.4%
Number of cigarettes per day (mean ± SD) [*n*]	9.1 ± 5.5
Max. number of cigarettes per day [*n*]	20
Min. number of cigarettes per day [*n*]	1
Regular consumption of cigarettes for how many years (mean ± SD) [years]	15.5 ± 4.7
Longest period [years]	23
Shortest period [years]	2
Time last cigarette was consumed (mean ± SD) [hours]	16.9 ± 34.2
Longest period [hours]	168
Shortest period [hours]	1
Group 3 E-cigarettes and/or hookahs [*n*]	42
E-liquid nicotine strength (mean ± SD) [mg/ml]	2.9 ± 4.1
Time last e-cigarette was consumed (mean ± SD) [hours]	14.4 ± 14.8
Longest period [hours]	48
Shortest period [hours]	2

SD, Standard deviation.

In Group 2 (smokers), 48 out of 61 patients consumed exclusively classic cigarettes (78.7%), ten out of 61 patients classic cigarettes and also hookahs (16.4%), and three out of 61 patients consumed classic cigarettes and are regularly vaping e-cigarettes (4.9%). For all smokers, the mean number of cigarettes consumed per day was 9.1 ± 5.5 (± SD) and the last cigarette was consumed on average 16.9 ± 34.2 h before oocyte pick-up. The patients included in Group 2 had smoked regularly for an average of 15.5 ± 4.7 years, whereby the longest period was 23 years and the shortest period 2 years. Patients vaping e-cigarettes in Group 3 consumed e-liquids with an average nicotine strength of 2.9 ± 4.1 mg/ml and the last e-cigarette was consumed on average 14.4 ± 14.8 h before oocyte pick-up.

### Nicotine, cotinine, and benzo[a]pyrene content in the follicular fluid

**Figure 1:**
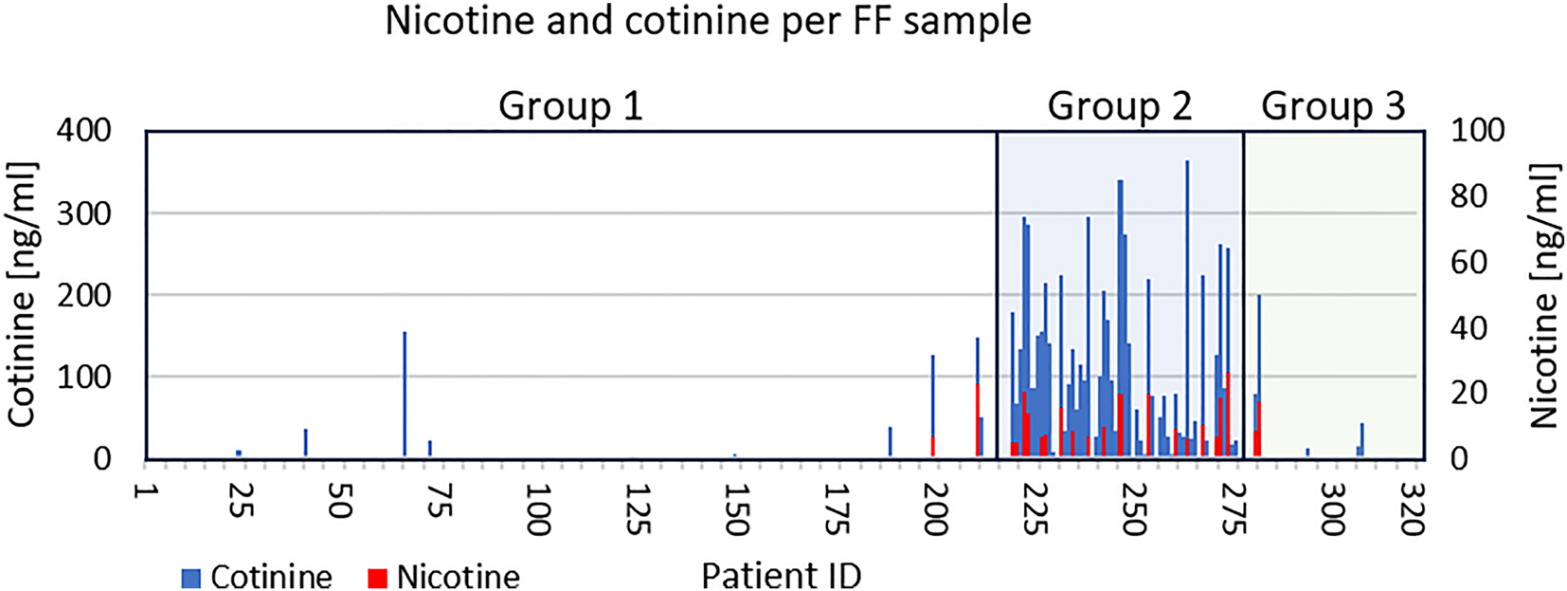
Nicotine and cotinine distribution for all 320 FF samples analysed—separated into Group 1 (non-smokers), Group 2 (smokers), and Group 3 (e-cigarettes and/or hookahs). Each bar represents one sample. Nicotine (red) and cotinine (blue) values in ng/ml FF. x axis represents patient IDs from 1 to 320, the left y axis the cotinine concentration (ng/ml) and the right y axis the nicotine concentration (ng/ml).

GC–MS was used to analyse FF samples from all 320 patients. LOD were 0.33 ng/ml for nicotine, 0.27 ng/ml for cotinine, and 0.03 ng/ml for benzo[a]pyrene. LOQ were 1.28 ng/ml for nicotine (linearity: 5–800 ng/ml), 1.04 ng/ml for cotinine (linearity: 5–800 ng/ml), and 0.11 ng/ml for benzo[a]pyrene (linearity: 0.5–80 ng/ml). The analyte distribution for all samples is shown in Fig. 1.

In Group 1 (non-smokers), nicotine was detected in two (0.9%) and cotinine in nine (4.1%) out of 217 FF samples. The average value was 0.1 ± 1.6 ng/ml (± SD) (maximum: 22.8 ng/ml) for nicotine and 3.0 ± 17.6 ng/ml (maximum: 155 ng/ml) for cotinine (see Table 3). Two of the patients with nicotine or cotinine in their FF had quit smoking months previously and four were passive smokers. Three of the nine patients with nicotine or cotinine in their FF from Group 1 did not smoke, had never smoked, or were passive smokers according to the questionnaire.

**Table 3:** Detection of nicotine, cotinine, and benzo[a]pyrene in the FF in Group 1 (non-smokers), Group 2 (smokers), and Group 3 (e-cigarettes and/or hookahs).

	Group 1 Non-smokers		Group 2 Smokers			Group 3 E-cigarettes and/or hookahs			
					*p* compared to Group 1			*p* compared to Group 1	*p* compared to Group 2
Samples [*n*]	217		61			42			
Detection of nicotine [*n*]/[%]	2	0.9%	18	29.5%	< 0.001^b^	2	4.8%	n.s.^b^	< 0.01^b^
Mean nicotine ± SD [ng/ml]	0.1	± 1.6	3.6	± 6.6	< 0.001^a^	0.6	± 2.9	n.s.^a^	< 0.01^a^
Max. value nicotine [ng/ml]	22.8		26.3			17.5			
Detection of cotinine [*n*]/[%]	9	4.1%	51	83.6	< 0.001^b^	5	11.9%	n.s.^b^	< 0.001^b^
Mean cotinine ± SD [ng/ml]	3.0	± 17.6	103.3	± 100.7	< 0.001^a^	8.4	± 33.4	n.s.^a^	< 0.001^a^
Max. value cotinine [ng/ml]	155.0		363.0			200.0			
Detection of benzo[a]pyrene [*n*]/[%]	0	0	0	0	–	0	0	–	–
Mean benzo[a]pyrene ± SD [ng/ml]	–	–	–	–	–	–	–	–	–
Max. value benzo[a]pyrene [ng/ml]	–	–	–	–		–	–		

Statistical analysis was performed using the ^a^Mann-Whitney U Test and ^b^Fisher's exact test between Group 2 and Group 1, between Group 3 and Group 1 and Group 3 and 2, respectively.

SD, standard deviation; n.s., not significant.

In Group 2 (smokers), nicotine and cotinine were detected at a significantly higher proportion compared with non-smokers (*p* < 0.001). Nicotine was present in 18 (29.5%) and cotinine in 51 (83.6%) out of 61 FF samples. The average value was 3.6 ± 6.6 ng/ml (maximum: 26.3 ng/ml) for nicotine and 103.3 ± 100.7 ng/ml (maximum: 363 ng/ml) for cotinine. Both average values were significantly higher compared with those of non-smokers (*p* < 0.001) (see Table 3).

In patients who exclusively consumed e-cigarettes and/or hookahs (Group 3), nicotine was detected in two (4.8%) and cotinine in five (11.9%) out of 42 FF samples. The average value was 0.6 ± 2.9 ng/ml (maximum 17.5 ng/ml) for nicotine and 8.4 ± 33.4 ng/ml (maximum 200.0 ng/ml) for cotinine (Table 3). Thus, nicotine and cotinine were detected for the first time in the FF of patients who exclusively consumed e-cigarettes and/or hookahs. Although not significantly different, the nicotine/cotinine concentrations in the FF and mean values in this group were three to six-fold higher compared with those of non-smoking patients.

Nicotine (*p* < 0.01) and cotinine (*p* < 0.001) were detected at a significantly lower proportion in patients who exclusively consumed e-cigarettes and/or hookahs compared to smokers (Group 2). Additionally, mean values for nicotine (*p* < 0.01) and cotinine (*p* < 0.001) were significantly lower in Group 3 compared to Group 2.

In all groups, a robust correlation was observed between the detection of nicotine and cotinine in individual FF samples (Fig. 1; *r* = 0.76; *p* < 0.001). Unsurprisingly, cotinine levels were much higher due to nicotine’s shorter half-life (~ two hours) compared with that of cotinine (~ 16 h) (mean nicotine: 12.5 ± 6.6 ng/ml; mean cotinine 203.9 ± 83.6 ng/ml).

Finally, although a very sensitive LOD (0.03 ng substance/ml FF) was achieved, benzo[a]pyrene was not detected in any of the 320 FF samples.

### Clinical parameters

No significant differences between groups were found regarding the average number of oocytes per OPU (Group 1: 8.9; 95%CI: 8.5–9.3; Group 2: 9.3; 95%CI: 8.6–9.9; Group 3: 9.5; 95%CI: 8.8–10.1) and number of mature MII per oocyte (Group 1: 6.5; MII Rate 73.0%; 95%CI: 67.9–79.6; Group 2: 6.8; MII Rate 73.1%; 95%CI: 61.9–84.2; Group 3: 6.8; MII Rate 71.6%; 95%CI: 57.9–85.2) (Table 4). The numbers of OPUs without oocytes or immature oocytes only, cycles without fertilisation, and cycles with irregular fertilisation were comparable and did not statistically differ between groups (see Table 4 for details). Fertilisation rates were 60.0% (95%CI: 53.8–66.8) in Group 1, 64.7% (95%CI: 52.7–76.7) in Group 2, and 60.3% (95%CI: 45.5–75.1) in Group 3. Rates of embryo transfers per OPU were not statistically different between non-smokers (91.7%; 95%CI: 88.1–95.3), smokers (90.2%; 95%CI: 82.8–97.7), and patients who exclusively consumed e-cigarettes and/or hookahs (85.7%; 95%CI: 75.1–96.3).

**Table 4:** Clinical parameters of all 320 patients are presented as mean with 95% confidence intervals (95%CI).

	Group 1 Non-smokers		Group 2 Smokers			Group 3 E-cigarettes and/or hookahs		
		95%CI		95%CI	*p*		95%CI	*p*
OPU [*n*]	217		61			42		
Oocytes per OPU [Ø]	8.9	8.5–9.3	9.3	8.6–9.9	n.s.^a^	9.5	8.8–10.1	n.s.^a^
Metaphase II oocytes per OPU [Ø]	6.5		6.8			6.8		
% MII/oocyte	73.0	67.9–79.6	73.1	61.9–84.2	n.s.^a^	71.6	57.9–85.2	n.s.^a^
2PN per OPU [Ø]	3.9		4.4			4.1		
% 2PN/MII	60.0	53.8–66.8	64.7	52.7–76.7	n.s.^a^	60.3	45.5–75.1	n.s.^a^
0PN per OPU [Ø]	2.1		1.9			2		
% 0PN/MII	32.3	25.8–38.2	27.9	16.6–39.1	n.s.^a^	29.4	15.6–43.2	n.s.^a^
≥ 3PN per OPU [Ø]	0.5		0.5			0.7		
% ≥ 3PN/MII	7.7	4.1–11.1	7.4	1.1–13.9	n.s.^a^	10.3	0–14.7	n.s.^a^
OPUs [*n*]	217		61			42		
OPUs without oocytes or MII [*n*]	7		3			1		
ICSI treatments [*n*]	210		58			41		
% ICSI/OPU	96.8	94.4–99.1	95.1	89.6–100	n.s.^b^	97.6	92.9–100	n.s.^b^
ICSI without or irregular fertilisation	11		3			5		
Embryo transfer [*n*]	199		55			36		
% ET/OPU	91.7	88.1–95.3	90.2	82.8–97.7	n.s.^b^	85.7	75.1–96.3	n.s.^b^
Clinical pregnancy [*n*]	88		22			17		
% Clinical pregnancy/OPU	0.6	34.0–47.1	36.1	24.1–48.1	n.s.^b^	40.5	25.6–55.3	n.s.^**b**^

OPU, Oocyte pick-up; PN, pronuclei; ISCI, intracytoplasmic sperm injection; n.s., not significant.

^a^Mann-Whitney U Test and ^b^Fisher's exact test were applied for clinical parameters.

The number of previous cycles, gonadotropin dosages, average days of embryo transfer, and average number of transferred embryos were not statistically different between groups (Tables 1 and 4). Clinical pregnancy rates per OPU were 40.6% (95%CI: 34.0–47.1) in Group 1, 36.1% (95%CI: 24.1–48.1) in Group 2, and 40.5% (95%CI: 25.6–55.3) in Group 3. Overall, MAR outcomes in non-smoking patients, smoking patients, and patients who exclusively consumed e-cigarettes and/or hookahs were comparable for all parameters, with no significant differences.

### Correlation analysis

**Figure 2:**
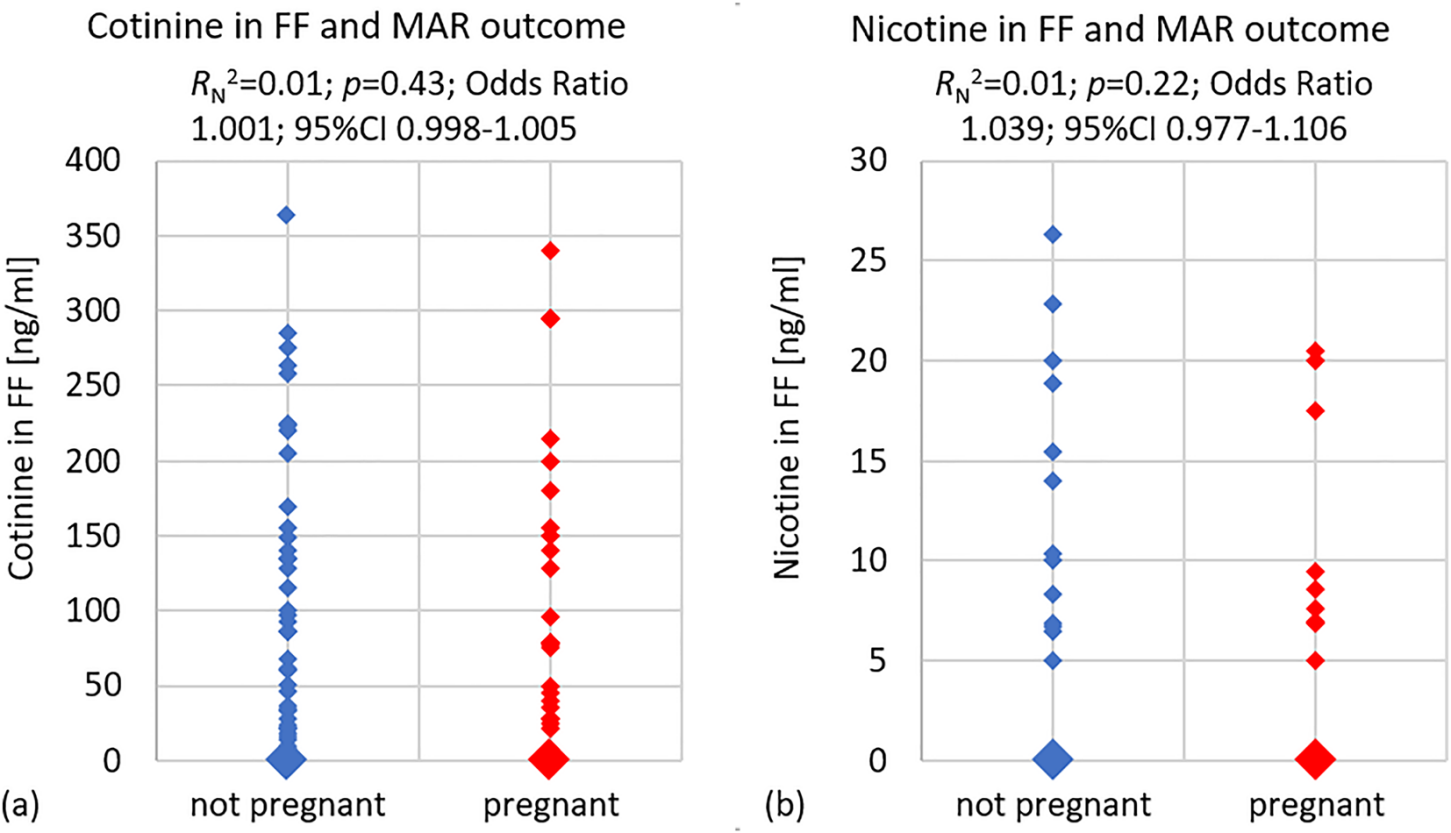
Binary logistic regression analysis with correlation factor *R*_N_^2^ for (**a**) cotinine contamination in the FF (ng/ml; left panel) and MAR outcome defined as not pregnant/pregnant and (**b**) nicotine contamination in the FF (ng/ml; right panel) and MAR outcome defined as not pregnant/pregnant. Each square represents one FF sample (*n* = 320)—a larger square indicates several samples with the same concentration for cotinine or nicotine, respectively.

Single and multiple linear and logistic correlation analyses were performed using data from all 320 samples analysed to assess the impact of nicotine and cotinine in the FF on MAR outcomes. No significant correlation was identified using single binary logistic regression analysis between cotinine concentrations in FF and clinical pregnancy (*R*_N_^2^ = 0.01; *p* = 0.43; odds ratio 1.001; 95%CI: 0.998–1.005) (Fig. 2a; left panel).

Similar results were obtained for nicotine (*R*_N_^2^ = 0.01; *p* = 0.22; odds ratio 1.039; 95%CI: 0.977–1.106) (Fig. 2b; right panel), as well as with multiple binary logistic regression analyses incorporating both nicotine and cotinine concentrations (*R*_N_^2^ = 0.01; *p* = 0.46; cotinine coefficient -0.001; odds ratio 0.999; 95%CI: 0.993–1.001 and nicotine coefficient 0.048; odds ratio 1.049; 95%CI: 0.951–1.158).

Using single linear regression models, no correlations were found for the number of oocytes per OPU, maturation and fertilisation rates, and cotinine or nicotine concentrations in FF. For cotinine, *r* values were 0.09 for the number of oocytes per OPU (*p* = 0.09), 0.04 for the maturation rate (*p* = 0.45), and 0.01 for the fertilisation rate (*p* = 0.83). Similar results were found for nicotine and the number of oocytes per OPU (*r* = 0.11; *p* = 0.08), the maturation rate (*r* = 0.06; *p* = 0.31), and the fertilisation rate (*r* = -0.02; *p* = 0.76).

Using multiple linear regression models with combined cotinine and nicotine concentrations in the FF, *R*^2^ values were 0.02 for the number of oocytes per OPU (*p* = 0.39), 0.01 for the maturation rate (*p* = 0.63), and 0.01 for the fertilisation rate (*p* = 0.88). Overall, no correlation was detected between the presence of nicotine and cotinine in the FF and MAR outcomes.

Additionally, patients’ questionnaires were evaluated to correlate consumption behaviour and the presence of nicotine and cotinine in the FF of smoking patients (*n* = 61; Group 2). There was a significant multiple variant correlation between the number of cigarettes consumed per day (*X*_1_), the time the last cigarette was consumed (*X*_2_), and cotinine concentrations in the FF (*R*^2^ = 0.07; *p* = 0.03). This correlation was only found between the number of cigarettes consumed per day and cotinine concentrations in the FF (*r* = 0.27; *p* = 0.03), while the time the last cigarette was consumed did not have a significant impact when considered separately (*r* = -0.21; *p* = 0.11).

No significant multiple or single correlations were found between nicotine concentrations and consumption behaviour. There was no significant correlation for the number of cigarettes consumed per day (*r* = 0.21; *p* = 0.11), the time the last cigarette was consumed (*r* = -0.08; *p* = 0.53), or for the multiple regression analysis taking both variables into account (*R*^2^ = 0.04; *p* = 0.39).

## Discussion

In this study, the accumulation of nicotine, cotinine, and benzo[a]pyrene was analysed in the FF of 320 women subjected to ICSI treatment due to male subfertility. Via a questionnaire, patients’ consumption behaviour was assessed to identify *i*) non-smoking patients, *ii*) patients who consumed classic cigarettes (with or without additional consumption of e-cigarettes and/or hookahs), and *iii*) patients with exclusive consumption of e-cigarettes or hookahs. To the best of our knowledge, this is the first time nicotine, cotinine, and benzo[a]pyrene have been quantified in the FF of patients exclusively vaping e-cigarettes or smoking hookahs.

The accumulation of cotinine or nicotine in the FF or ovarian tissue in smoking patients undergoing MAR treatment has been reported^8,25,38–42^. In the present study, concentrations of nicotine and cotinine were comparable to previous studies. The accumulation of nicotine and cotinine in individual FF samples presented a strong correlation, as well as patient consumption behaviour and cotinine concentrations in the FF—which underlines the significance of the questionnaire.

In a Canadian study by Neal et al.^43^, benzo[a]pyrene levels of 1.32 ± 0.68 ng/ml were detected in the FF of 19 smokers using GC–MS analysis. In another Canadian study by Zenzes and colleagues^25^, benzo[a]pyrene-DNA adducts were quantified via immunostaining granulosa cells of patients undergoing MAR treatment.

In this study, we did not detect benzo[a]pyrene in any of the 320 samples analysed, even though a very sensitive LOD of 0.03 ng benzo[a]pyrene/ml FF was applied. However, benzo[a]pyrene concentrations per cigarette vary between tobacco brands and also within one brand and different consumer regions/countries. Additionally, the cigarette production process and cigarette smoke composition have gradually changed during the last decades^44–46^. For example, Yershova et al.^44^ analysed benzo[a]pyrene in 43 different tobacco brands, reporting levels varying between 4 and 44 ng/cigarette. Hence, different cigarette compositions and consumer regions might explain the lack of benzo[a]pyrene detection in the FF of the German women in this study.

For the first time, nicotine and cotinine were detected in the FF of patients exclusively vaping e-cigarettes or smoking hookahs. The frequency and concentrations of nicotine and cotinine were significantly lower compared with smoking patients. However, it should be noted that the average nicotine strength of the consumed e-liquids according to the questionnaire can be considered as low (2.9 ± 4.1 mg nicotine per ml), no data about the daily consume of e-cigarettes were available and Group 3 was heterogeneous (regular/occasional hookah consumers with or without regular/occasional vaping). Taken together, this could explain the lower values ​​of nicotine and cotinine in the FF of patients exclusively vaping e-cigarettes or smoking hookahs compared to smokers. Nevertheless, nicotine and cotinine were found more frequently in the FF and mean values were increased in Group 3 compared with non-smoking patients. Vaping liquids, even those that are nicotine-free, and hookah tobaccos contain or produce several potentially harmful substances. These include propylene glycol, glycerine, formaldehyde, acetaldehyde, methylglyoxal, benzaldehyde, and endocrine-disrupting chemicals^28–30^. The cytotoxicity and, in particular, ovarian toxicity of e-cigarette liquids were demonstrated in animal models, in vitro and in vivo. Investigations in humans are rare and overall knowledge regarding the impact of vaping on human fertility is limited^47^. Nevertheless, in vitro exposure of ovaries to propylene glycol and glycerine leads to ovarian dysfunctionality, which includes morphological damage, oxidative balance disruption, and an increased apoptosis rate in rats^48^. In vitro exposure of ovaries to e-cigarette liquids in rodents impairs folliculogenesis and oestrogen secretion^49^; additionally, it affects mitochondrial function and the proteome in Chinese hamster V79 lung fibroblast cell lines by depletion of numerous proteins—especially related to mitochondrial functionality^50^. In vivo exposure to vaping substances also hampers metabolic functionality and gene expression in mice^51,52^. The potential adverse effects of heating liquids or smoking hookahs need to be examined in future studies with larger sample sizes—particularly in humans.

Smoking affects general health and is associated with several severe diseases. There is an ongoing debate about whether, and to what degree, smoking limits MAR outcomes. While some studies reported adverse effects with reduced clinical pregnancy rates^33,34^, increased abortion rates^33,53^, fewer oocytes per OPU^42,54–56^, lower fertilisation rates^54,57^, reduced endometrial thickness^37^, higher gonadotropin dosages for ovarian stimulation^58^, and increased cytoplasmic and meiotic anomalies^55,59^, others did not confirm these findings. In several studies, clinical pregnancy rates^37,38,53,54,57^, numbers of oocytes^37,59,60^, maturation rates^37,60^, fertilisation rates^38,60^, embryonic morphology and development^38,57^, implantation rates^57^, and miscarriage rates^37^ were similar between smoking and non-smoking patients undergoing MAR treatment.

In a systematic review including 21 studies^35^, patients who smoked had a significantly lower clinical pregnancy rate per cycle (OR 0.56, 95%CI: 0.43–0.73) and a significantly higher spontaneous miscarriage rate (OR 2.65, 95%CI: 1.33–5.30). However, the sample sizes of the studies included were low and varied between 41 and 834 cycles for clinical pregnancy rates (overall *n* = 5243) and between 23 and 1196 for miscarriage rates (*n* = 1,899). In contrast, in a recent retrospective cohort study on assisted reproductive technology (ART) cycles from the United States, nearly 750,000 ART cycles between 2009 and 2013 were analysed for MAR outcomes in smoking and non-smoking subjects^36^. The number of cycle cancellations (cycles halted before egg retrieval or embryo transfer) was increased in smoking patients; however, the clinical pregnancy, live birth, and miscarriage rates were comparable between non-smokers and smokers^36^.

It was hypothesised that smoking is particularly harmful for older patients^60^. In our study, no correlation between smoking, age, and MAR outcomes was found, nevertheless, it should be noted that our patient cohort was relatively young (mean 33.0 ± 3.6 years); only 22 patients were ≥ 39 years old. Smoking reactants and products can interfere with hormonal and metabolic homeostasis, cellular growth, and genetic constitution during folliculogenesis and oogenesis, as shown in several studies in vitro and in vivo^24,58,60–64^. Negative selection might eliminate impaired germ cells during hormonal stimulation so that only capable oocytes develop. Consequently, these oocytes possess a normal capacity for maturation, fertilisation, and embryonic development. In patients of advanced age, the oocyte pool might already be compromised and, likewise, the possibility of obtaining healthy oocytes with regular potential for maturation, fertilisation, and embryonic development. Thus, negative effects due to smoking do not necessarily affect MAR outcomes in patients undergoing ICSI treatment – especially younger patients.

In this study, the outcome of the MAR treatment was not negatively correlated with smoking. It should be noted that smoking not only impairs female gametogenesis and natural conception, but also has adverse effects on male spermatogenesis. The influence of smoking on sperm quality and quantity, as well as epigenetic alterations, has been described in several studies^74–78^. Accordingly, smoking itself could be a decisive factor for the origin of male infertility and thus one reason for MAR treatment. As shown in this study, MAR Treatment may overcome natural limitation associated with smoking. However, transgenerational and thus long-lasting effects of smoking to the offspring—especially with regards to epigenetic factors—cannot be excluded after ICSI treatment to overcome these limitations^79,80^. Without doubt, further studies are necessary to assess the risks of smoking and vaping on potential transgenerational effects after MAR treatment.

Furthermore, the quantity of smoking is a relevant factor. Many studies do not discriminate between regular smokers and those with the occasional consumption of tobacco products. In the present study, consumption behaviour was assessed and the correlation between consumption behaviour, individual FF levels of smoking toxins, and MAR outcomes was analysed. No negative effect was observed between cotinine and nicotine levels in the FF and number of oocytes, maturation rates, fertilisation, or clinical pregnancy rates. However, although this study includes one of the largest prospective datasets with at least 320 patients undergoing MAR treatment, smoking products were detected in only one-third of the samples analysed. Further studies with larger sample sizes—especially for vaping or hookah-smoking patients—are necessary to assess the impact of smoking/vaping on MAR treatment outcomes.

## Conclusion

In the present study, we demonstrated that nicotine and cotinine can be quantified in the human FF of patients undergoing MAR treatment, while benzo[a]pyrene was not detectable in the 320 samples analysed. Smoking status and individual accumulation of smoking toxins in the FF had no statistically significant impact on MAR treatment outcomes. Clinical pregnancy, maturation, and fertilization rates and the numbers of retrieved oocytes were not statistically significantly different. Patient consumption behaviour was evaluated via a questionnaire and, for the first time, nicotine and cotinine were quantified in the FF of patients exclusively vaping e-cigarettes or smoking hookahs. Regarding MAR outcomes, no statistically significant adverse effects were found in patients who vaped or smoked hookahs. The potential adverse effects of vaping liquids or smoking hookahs need to be examined in further studies with larger sample sizes.

### Supplementary Information

Supplementary Information 1.

Supplementary Table 1.

## Data Availability

All data generated or analysed in this study are included in this published article. The questionnaire used during this study is available from the corresponding author upon request.

## Abbreviations

MAR: Medically assisted reproduction
ICSI: Intracytoplasmic sperm injection
e-cigarette: Electronic cigarette
FF: Follicular fluid
GC–MS: Gas chromatography–Mass spectrometry
E2: Serum oestradiol
AMH: Anti-Müllerian hormone
IU: International unit
MII: Metaphase II oocyte
0PN: Oocyte without pronuclei
1PN: One pronucleus oocyte
2PN: Two pronuclei oocyte
3PN: Three or more pronuclei oocyte
BMI: Body mass index
OPU: Oocyte pick-up
LOD: Limits of detection
LOQ: Limits of quantification
SD: Standard deviation
95%CI: 95% Confidence interval
