# Supplementary material for: Consumption of hookahs, e-cigarettes, and classic cigarettes and the impact on medically assisted reproduction treatment
Source: Sci Rep. 2024 Apr 26;14:9597. doi: 10.1038/s41598-024-60251-y (PMC11053167; doi:10.1038/s41598-024-60251-y)
Supplement: Supplementary file 1 — Supplementary Information 1. [file 41598_2024_60251_MOESM1_ESM.docx]

**Patient questionnaire**

We ask you to answer the following questions as completely as possible. If you do not wish to comment on some questions, please leave the respective question(s) unanswered.

Data protection notice:

The data collected using this questionnaire will be used confidentially and exclusively anonymously and will only be analyzed for research purposes and statistical classification. Your data will neither be used commercially nor passed on to third parties.

1. Do you currently smoke classic cigarettes?

- Yes, regularly
- Yes, occasionally (i.e. less than one cigarette per day on average)
- No

1. How many cigarettes do you smoke on average per day?

- Number of cigarettes: ˾__˾__˾__
- On average, less than one cigarette per day

1. How many hours ago did you smoke your last cigarette?

˾__˾__ hours

1. What brand of cigarettes or tobacco do you smoke mainly, and what other brands?

(*Please state the exact name, including additional information such as “Medium”, “Light”, “Ultra”, “with/without filter”, etc.*)

- Mainly: ˾___________________________
- Also: ˾___________________________
- Also: ˾___________________________

1. Do you currently smoke or have you ever smoked cigars/cigarillos or pipes?

- No
- Yes, before, but not anymore
- Yes, currently, but only occasionally (less than one per day)
- Yes, regularly

1. At what age did you start smoking regularly/occasionally?

Age in years: ˾__˾__

1. Have you ever smoked cigarettes before (regularly or occasionally)?

- Yes, regularly Number of cigarettes per day: ˾__˾__˾__
- Yes, occasionally (less than one cigarette per day on average)
- No, never

1. And how long has it been since you smoked cigarettes?

- Since month
- Since years

1. Does your partner smoke or has smoked for a long period of time?

- No
- Yes, before, but not anymore
- Yes, currently, but only occasionally (less than one per day)
- Yes, regularly at the moment

1. Do you currently smoke or have ever smoked Hookahs?

- No
- Yes, before, but not anymore
- Yes, currently, but only occasionally (less than once a day)
- Yes, regularly at the moment

1. Do you currently smoke or have ever smoked e-cigarettes?

- No
- Yes, before, but not anymore
- Yes, currently, but only occasionally (less than once a day)
- Yes, regularly at the moment

1. If you currently regularly use e-cigarettes, what nicotine strength do you use?

Nicotine strength: ˾__˾__ mg/ml

1. How many hours ago did you last use an e-cigarette?

˾__˾__ hours
