# Supplementary material for: Consumption of hookahs, e-cigarettes, and classic cigarettes and the impact on medically assisted reproduction treatment
Source: Sci Rep. 2024 Apr 26;14:9597. doi: 10.1038/s41598-024-60251-y (PMC11053167; doi:10.1038/s41598-024-60251-y)
Supplement: Supplementary file 2 — Supplementary Table 1. [file 41598_2024_60251_MOESM2_ESM.docx]

Supplementary Table I. Retention times (*t*_R_), target, and qualifier ions for cotinine, nicotine, and benzo[a]pyrene used for GC-MS.

| Parameter | *t*_R_  (min) | Target ion (m/z) | Qualifier ion I (m/z) | Qualifier ion II (m/z) |
| --- | --- | --- | --- | --- |
| Cotinine | 6.70 | 176 | 98 |  |
| Cotinine-D_3_ | 6.70 | 179 | 101 |  |
| Nicotine | 5.46 | 162 | 133 | 161 |
| Nicotine-^13^CD_3_ | 5.45 | 166 | 137 | 88 |
| Benzo[a]pyrene | 9.36 | 252 | 250 |  |
| Benzo[a]pyrene-D_12_ | 9.35 | 264 | 263 |  |
